# Long-Term Survival and Value of ^18^F-FDG PET/CT in Patients with Gastroenteropancreatic Neuroendocrine Tumors Treated with Second Peptide Receptor Radionuclide Therapy Course with ^177^Lu-DOTATATE

**DOI:** 10.3390/life11030198

**Published:** 2021-03-04

**Authors:** Margarida Rodrigues, Kevin-Klaus Winkler, Hanna Svirydenka, Bernhard Nilica, Christian Uprimny, Irene Virgolini

**Affiliations:** Department of Nuclear Medicine, Medical University of Innsbruck, Anichstrasse 35, 6020 Innsbruck, Austria; kevin.winkler@student.i-med.ac.at (K.-K.W.); hanna.svirydenka@tirol-kliniken.at (H.S.); bernhard.nilica@tirol-kliniken.at (B.N.); christian.uprimny@tirol-kliniken.at (C.U.); irene.virgolini@tirol-kliniken.at (I.V.)

**Keywords:** neuroendocrine tumors, peptide receptor radionuclide therapy, ^177^Lu-DOTATE, ^18^F-FDG PET

## Abstract

Peptide receptor radionuclide therapy (PRRT) has been recognized as a promising therapy against neuroendocrine tumors (NETs). The use of ^18^F-fluorodeoxyglucose (^18^F-FDG) positron emission tomography (PET) in NETs has been a matter of controversy. The purpose of this study was to evaluate the long-term survival and efficacy of a second PRRT course with ^177^Lu-DOTATE in patients with advanced gastroenteropancreatic (GEP) NETs. Furthermore, the value of ^18^F-FDG PET/CT in these patients was evaluated. 40 patients with GEP NETs who underwent two PRRT courses with ^177^Lu-DOTATATE and combined examinations with ^68^Ga-DOTA-TOC and ^18^F-FDG PET/CT were evaluated. After the second PRRT course, two patients (5.0%) were in partial remission, 21 patients (52.5%) in stable disease and 17 patients (42.5%) had progressive disease. The median overall survival was 122.10 months. After the second PRRT course, the median overall survival was significantly higher (*p* = 0.033) in the ^18^F-FDG-negative group compared to the ^18^F-FDG-positive group (145.50 versus 95.06 months, respectively). The median time to progression was 19.37 months. In conclusion, a second PRRT course with ^177^Lu-DOTATE is an effective treatment approach for GEP NET patients with disease progression. A change in ^18^F-FDG status after PRRT may predict the disease course and survival. Patients who are ^18^F-FDG-negative have a significantly longer overall survival than those who are ^18^F-FDG-positive.

## 1. Introduction

Neuroendocrine tumors (NETs) are a heterogeneous group of neoplasms characterized by their overexpression of somatostatin receptors on the cell surface [1,2]. The incidence of NETs has been rising over the past 30 years, particularly those arising from the midgut and pancreas [3]. The World Health Organization (WHO) guidelines classify gastroenteropancreatic (GEP) NET into three grades: low grade (grade 1, Ki-67 < 2%), intermediate grade (grade 2, Ki-67 3%–20%) and high grade (grade 3, Ki-67 > 20%) [4].

NETs can be asymptomatic for years and so they are often diagnosed in a metastatic or locally advanced state where a systemic therapy is required [5,6].

Somatostatin receptor (SR) imaging with ^68^Ga-labelled peptide positron emission tomography (PET)/computed tomography (CT) has been shown to provide excellent sensitivity and specificity for diagnosing and staging NETs [7,8]. Nilica et al. [9] showed that the investigation of only SR status by ^68^Ga-DOTA-TOC PET/CT may not reflect progression in a certain NET lesion which might induce an earlier therapy start or an alternative therapy.

^18^F-fluorodeoxyglucose (FDG) PET/CT is used to assess glycolytic metabolism, and a higher uptake of ^18^F-FDG has been found to be associated with tumor aggressiveness [10]. ^18^F-FDG PET/CT has thus been used increasingly in the past few years for the evaluation of high-grade NETs [10,11]. ^18^F-FDG PET is not indicated primarily for NETs because of its poor sensitivity to detect tumors with low metabolic activity and slow growth [12]. However, Binderup et al. [13] showed that although the diagnostic sensitivity of ^18^F-FDG PET is low for NETs, the prognostic value is high.

Peptide receptor radionuclide therapy (PRRT) is a molecularly targeted radiation therapy involving the systematic administration of a specific radiopharmaceutical composed of a β-emitting radionuclide chelated to a peptide, designed to target with high affinity and specificity receptors overexpressed on the cell surface of tumors. In general, PRRT is used after failing first-line medical therapy. The standard indication for PRRT is a metastatic and inoperable SR-positive NET, evaluated by SR imaging with PET or scintigraphy [14,15,16,17]. The high absorbed tumor dose following PRRT may lead to partial or even complete objective response. Gabriel et al. [18] reported that the documented overall response to treatment in GEP NETs, neuroectodermal tumors (such as paraganglioma or phaeochromocytoma) or lung NETs is ~80%, stable disease/minor response ~55%, complete/partial remission ~25% and progressive disease ~20%. PRRT has been shown to also be effective in terms of both symptomatic control and survival [15,18,19]. The NETTER-1 trial [19], a randomized, controlled international phase three study that evaluated the efficacy and safety of ^177^Lu-DOTATATE in patients with advanced progressive, SR-positive midgut NET, found that treatment with ^177^Lu-DOTATATE resulted in markedly longer progression-free survival and a significantly higher response rate than a high-dose of the long-acting, non-radioactive octreotide among these patients. The time to progression was significantly higher in patients treated with ^177^Lu-DOTATATE compared to that in patients treated with octreotide LAR, with a median time to progression not reached for ^177^Lu-DOTATATE and 8.7 months for octreotide LAR [19].

The purpose of this retrospective study was to evaluate the long-term survival and efficacy of a second PRRT course with ^177^Lu-DOTATE in patients with advanced GEP NETs. Furthermore, it aimed to evaluate the value of ^18^F-FDG PET/CT in these patients and whether possible changes in tumor ^18^F-FDG-uptake correlate with disease course.

## 2. Results

### 2.1. Survival 

**Figure 1:**
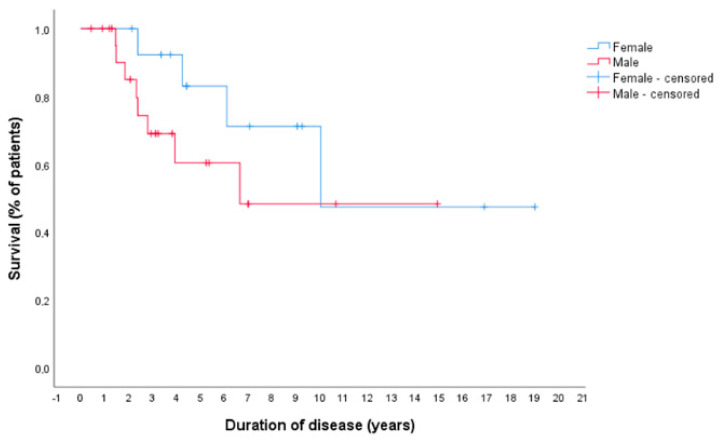
Life span curve.

The median overall survival was 122.10 months. The longest survival was 231 months in a 56-year-old female who is still alive. No significant difference in duration of disease was found between male and female patients (122.18 months versus 80.97 months, respectively; log-rank test *p* = 0.246) (Figure 1).

### 2.2. Disease Course after Second PRRT Course

At final evaluation, 28 (70.0%) patients (18 male and 10 female) were alive and 12 (30%) patients (eight male and four female) died. The shortest duration of NET evaluation after a second PRRT course was 30 days in a 54-year-old man, who died due to NET progression.

**Figure 2:**
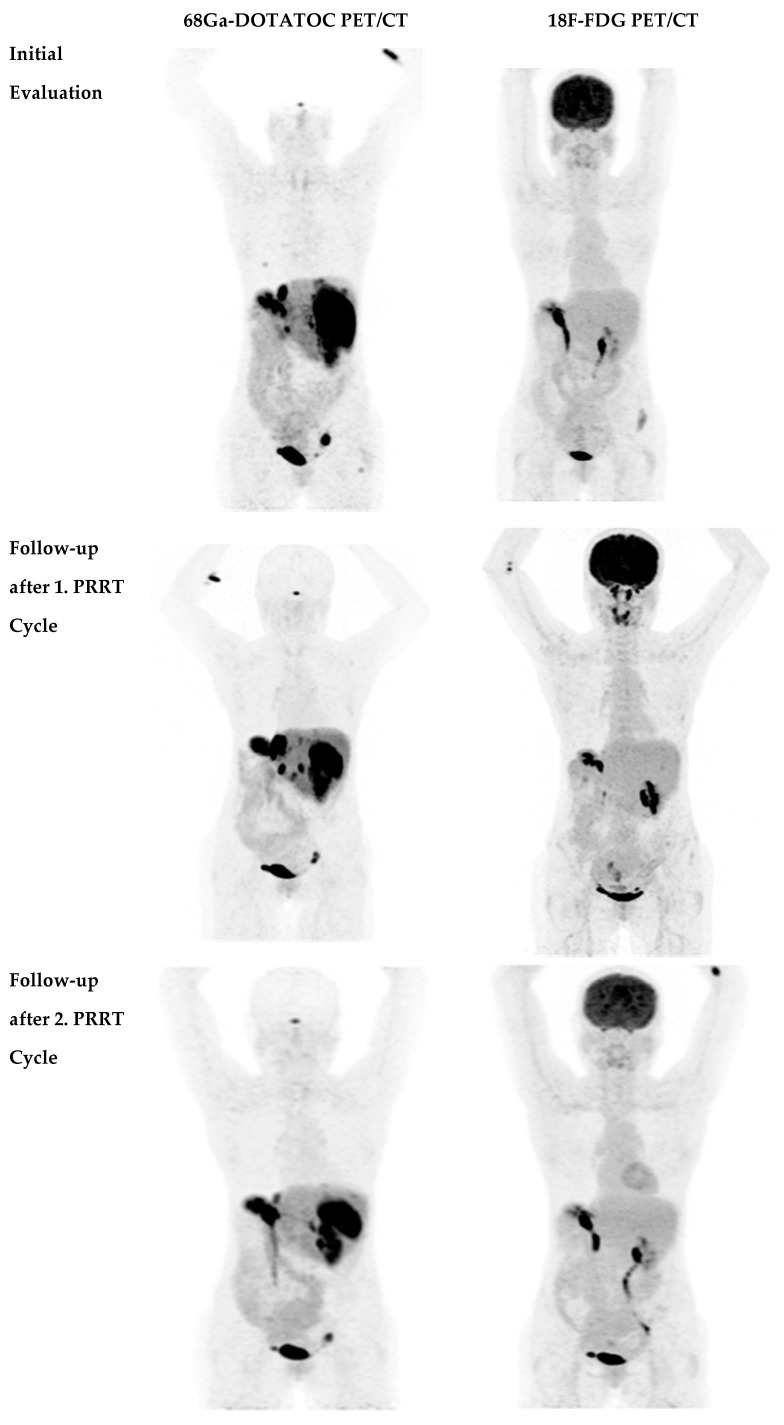
30year-old male patient with NET G2 in the pancreatic tail, Ki-67 12% in liver metastases, and right hepatectomy. ^68^Ga-DOTA-TOC and to a less extent ^18^F-fluorodeoxyglucose (^18^F-FDG) positron emission tomography (PET) show multiple liver metastases at baseline and partial remission after the second PRRT with ^177^Lu-DOTA-TATE. CT: computed tomography.

**Figure 3:**
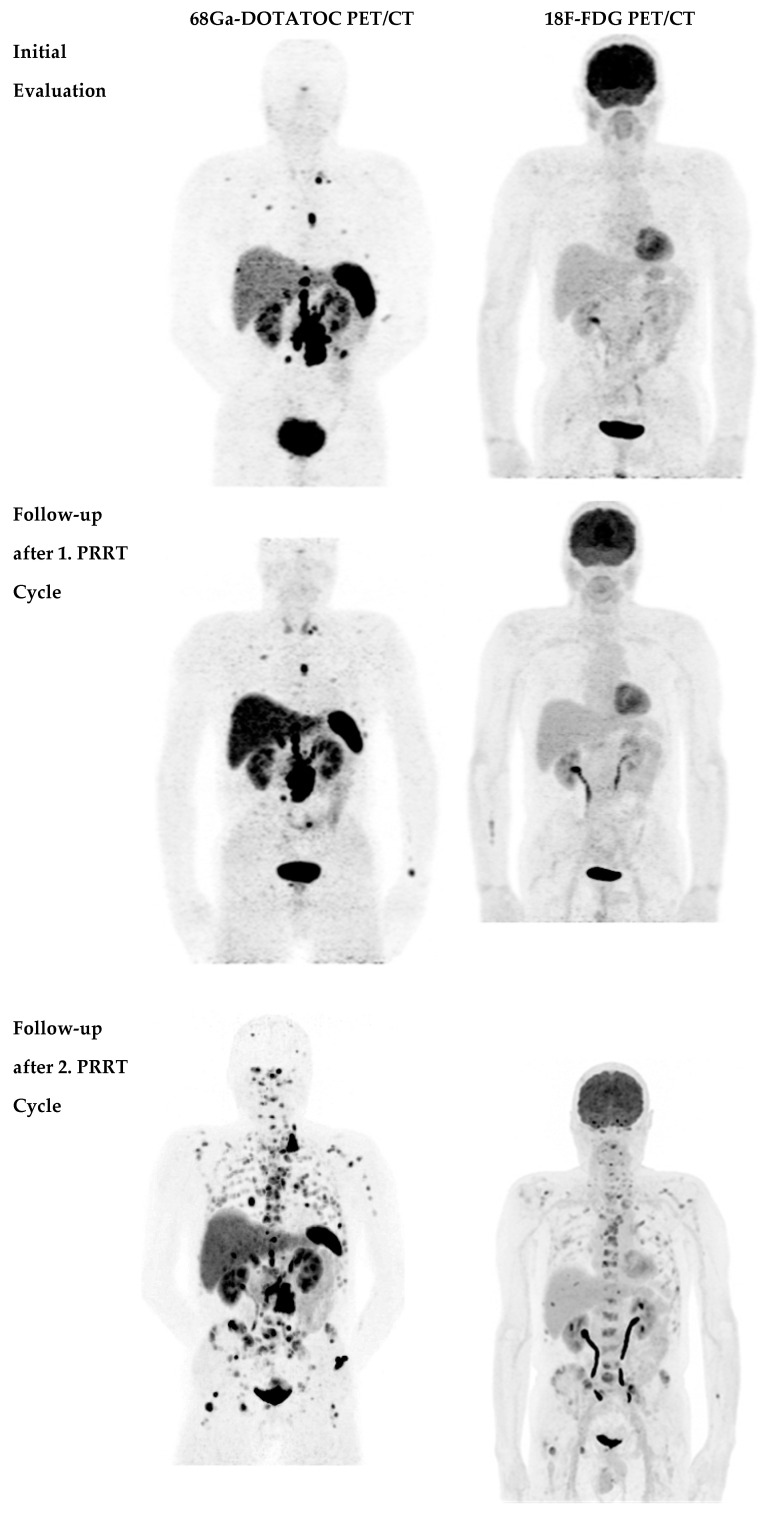
60-year-old male patient with NET G2 in the ileum. ^68^Ga-DOTA-TOC PET shows bone, lymph node and liver metastases at baseline, stable disease after 1 PRRT cycle, and progressive disease after the second PRRT with ^177^Lu-DOTA-TATE while ^18^F-FDG-PET was negative both at initial evaluation and after 1 PRRT cycle.

The disease course after the second PRRT course is listed in Table 1. Out of 40 patients, two patients (5.0%) were in partial remission (Figure 2), 21 patients (52.5%) were in stable disease and 17 patients (42.5%) had progressive disease (Figure 3).

**Figure 4:**
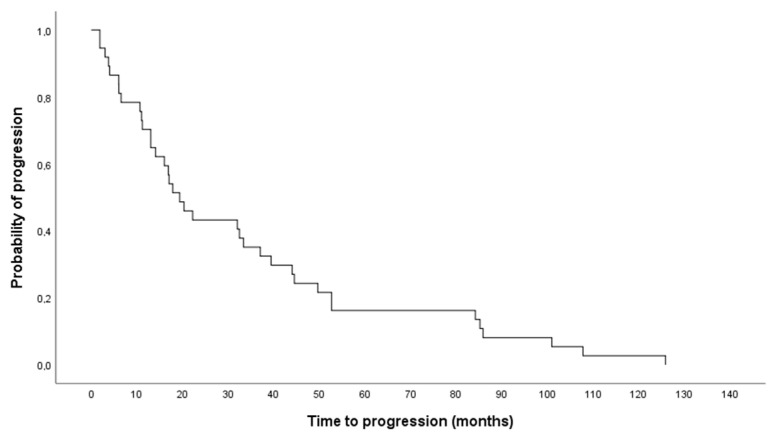
Time to progression after the second therapy course (n = 40 patients).

The median time to progression was 19.37 months (Q1–Q3, 10.83–47.07 months) (Figure 4). No significant difference in time to progression between male and female patients could be found (mean time to progression 26.06 ± 28.34 months versus 47.49 ± 37.37 months, respectively).

### 2.3. ^18^F-FDG Uptake Status Related to Survival

At baseline, 33 (82.5%) patients were ^18^F-FDG-negative, and 7 (17.5%) patients were ^18^F-FDG-positive. After the second PRRT course, 26 (65.0%) patients were ^18^F-FDG-negative, and 14 (35.0%) patients were ^18^F-FDG-positive (Table 2).

After the first PRRT course out of 40 patients, 35 (87.5%) patients have undergone no change in their ^18^F-FDG status and five (12.5%) patients changed their ^18^F-FDG status, with four (10.0%) patients from negative to positive and one (2.5%) patient from positive to negative ^18^F-FDG status. After the second PRRT course, out of 40 patients, 30 (75.0%) patients have undergone no change in their ^18^F-FDG status and 10 (25.0%) patients changed their ^18^F-FDG status, with seven (17.5%) patients from negative to positive ^18^F-FDG status and three (7.5%) from positive to negative ^18^F-FDG status.

**Figure 5:**
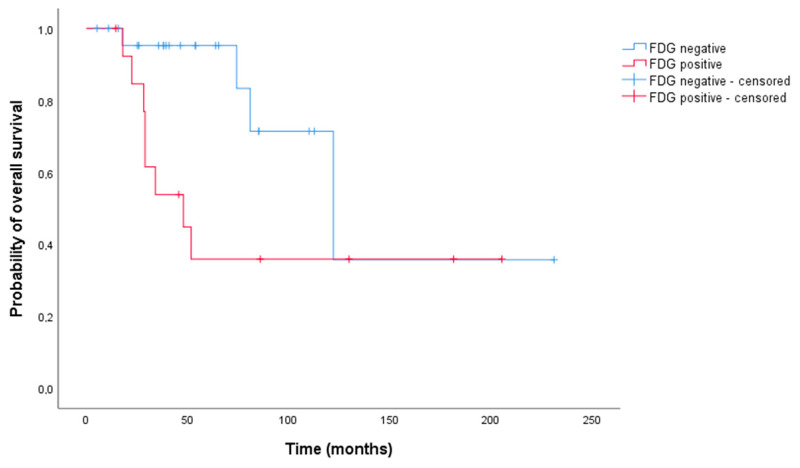
Kaplan–Meier curves of overall survival, ^18^F-FDG status after second PRRT course (Log-rank test, *p* = 0.033).

After the second PRRT course, the median overall survival was significantly higher (*p* = 0.033) in the ^18^F-FDG-negative group compared to the ^18^F-FDG-positive group. The median overall survival was 145.50 months (95% CI, 83.34–207.67) versus 95.06 months (95% CI, 48.36–141.77), respectively (Figure 5).

## 3. Discussion

PRRT has been increasingly recognized as one of the most promising therapies against NETs. Our study aimed to evaluate the long-term efficacy and survival after a second PRRT course with ^177^Lu-DOTATE. Overall, eight (30.77%) of 26 male and four (28.57%) of 16 female patients died, which means that nearly two thirds of patients were still alive after a second PRRT course with ^177^Lu-DOTATE. Recently, in a twelve-year follow-up after a first full PRRT course performed at our department, Gabriel et al. [18] found that 32% (14/44 patients) of the patients with metastatic or inoperable NET disease were still alive more than 12 years after the beginning of PRRT, with a median overall survival of 79 months. Other studies reported a median overall survival ranging from 22 to 71 months [15,20,21,22]. The longer median overall survival of 122.10 months found in our cohort of patients might be attributed to the second PRRT course performed in the event of disease progression. The median interval between histological diagnosis and the first scintigraphic examination in this study was 5.83 months (Q1–Q3, 1.40–26.98 months). A shorter interval might induce an earlier therapy start date and thereby result in a better prognosis. In roughly 20% of patients, toxicity findings (mild or moderate increase in nephro- or hematotoxicity grade) were observed. However, in terms of safety, re-PRRT had no critical impact on further oncologic treatment options in the case of disease progression (data in publication).

Although in our study no significant difference could be identified regarding a benefit for the female sex, as reported in other studies [18,23], the Kaplan–Meier survival plots seem to indicate a survival benefit with regard to the female sex. This benefit can be assumed as an inherent gender effect [18] because there was no significant difference in age, disease spread, diagnosis or therapeutic methods used in our cohort of patients. 

Although ^18^F-FDG PET is widely used in oncology, its use in NETs has been a matter of controversy. Binderup et al. [13] showed that a negative ^18^F-FDG-PET result is predictive of low aggressiveness and a high survival rate. Zhang et al. [24] reported a significant benefit in overall survival and in progression-free survival for their ^18^F-FDG-negative group of patients. We found similar results, where the median overall survival after the second PRRT cycle course was significantly longer in the ^18^F-FDG-negative group compared to the ^18^F-FDG-positive group. However, our study had two limitations. First, it used a retrospective design, and second, it assessed a relatively small number of patients in a single institution. Therefore, our results may have been affected by selection bias. 

In agreement with our previous report [9], we found in the present study that seven out of 40 patients developed ^18^F-FDG-positive lesions in the long term follow up, while we observed in three patients a change from ^18^F-FDG-positive status to ^18^F-FDG-negative status after the second PRRT course. This might conclude to a regression of tumor aggressiveness and a better survival for these patients. Accordingly, we found that the patients who underwent a change from their ^18^F-FDG-positive status into a ^18^F-FDG-negative status after the second PRRT course were still alive at the end of the study and had partial remission to stable disease. In contrast, four out of seven patients who underwent a change from their ^18^F-FDG-negative status into a ^18^F-FDG-positive status deceased under stable to progressive disease. This might be due to a higher tumor aggressiveness in the ^18^F-FDG-positive tumor lesions. Our results seem thus to confirm that a change in ^18^F-FDG status after PRRT may predict the disease course and survival.

Applying a dual-tracer approach with SR and ^18^F-FDG-PET imaging therefore can help the decision-making process for therapy selection in NET patients. In cases with a change of ^18^F-FDG status, rebiopsy to reevaluate the NET grade or Ki-67 might be considered.

PRRT is a well-established treatment for patients with GEP NET G1 and G2. In GEP NET G3 patients, the application of PRRT is still limited due to previous assumptions that because of lacking or low SR expression and rapid tumor growth rate, a benefit from PRRT should be not expected [25]. However, in recent retrospective studies [26,27] it has been shown that a substantial number of high-grade NETs showed an increased uptake on SR imaging, which could indicate a high tumor SR expression. Supporting the effectiveness of PRRT in GEP NET G3 with positive SR imaging, a recent review by Sorbye et al. [28] reported promising response rates (31–41%) and disease control rates (69–78%) in these tumors. In agreement with these results, we could confirm the value of PRRT in eight patients with GEP NET G3 included in this study.

Cytotoxic treatment with cisplatin and etoposide has been used in the past few years as a standard treatment for metastatic neuroendocrine carcinomas [29]. However, the progression-free survival after platinum-based chemotherapy is short (2.4–5 months) for NET G3 [26,27,28]. Therefore, a NET G2-like medical treatment strategy could be more appropriate for patients with NET G3 [28]. Therapeutic alternatives comprise temozolomide-based chemotherapy and PRRT. Further studies, as are on-going in our department, are needed to assess the value of PRRT/chemoradionuclide therapy for NET G3 and other aggressive NETs after the second PRRT course that show ^18^F-FDG-positive lesions.

In conclusion, our study shows that a second PRRT course with ^177^Lu-DOTATE is an effective treatment approach for GEP NET patients with disease progression. Furthermore, a change in ^18^F-FDG status after PRRT may predict disease course and survival. Patients who are ^18^F-FDG-negative have a significantly longer overall survival than those who are ^18^F-FDG-positive (median of 122.10 months versus 48.00 months in our cohort of patients).

## 4. Materials and Methods

### 4.1. Patients

A cohort of 40 patients with a histological confirmation of NETs (according to the European Neuroendocrine Tumors Society (ENETS) criteria) [4] who underwent both two PRRT courses with ^177^Lu-DOTATATE (after the confirmation of SR-positive lesions with ^68^Ga-DOTA-TOC PET/CT), and three combined studies with ^68^Ga-DOTA-TOC and ^18^F-FDG PET/CT at our department between 2005 and 2019 was retrospectively evaluated.

The local ethics committee approved the study (EK Nr: 1195/2018), and all patients gave their written informed consent.

The present evaluation included a total of 26 (65%) males and 14 (35%) females. The mean age at initial diagnosis was 54.63 ± 12.85 years, with the youngest patient being 29 years old and the oldest patient being 83 years old at the time of initial diagnosis. The majority (72.5%) of the 40 patients investigated had a well-differentiated NET of grade 2. The Ki-67 index was evaluated with immunohistochemistry. The demographic details are given in Table 3.

All patients included in the study were in advanced stages, requiring systemic antitumor therapy in a palliative setting and were in progressive disease after the first PRRT course. In particular, more than 55% of patients had metastases in more than one location, and most of them showed widespread metastases. Before undergoing PRRT, 16 patients were treated with other modalities including resection of the primary tumor (eight patients), radiofrequency ablation or embolization of metastases (one patient) or combined treatment with either resection, radiofrequency ablation or embolization of metastases (seven patients). The remaining 24 patients with widespread metastases were referred for PRRT without previous therapy.

The median overall survival, meaning the time after which 50% of patients were still living and 50% had died, included the time from the initial histological diagnosis or the first examination by ^68^Ga-DOTATOC PET/CT to the date of death or, for survivors, the last day of evaluation, namely, 31 December 2019. The median overall survival refers to how long patients survived with a NET disease. In particular, it was the time when half the patients were expected to be alive.

The time to progression was calculated as the time between first PRRT course and the second PRRT course. 

### 4.2. Peptide Receptor Radionuclide Therapy (PRRT) Regimen

The median interval between histological diagnosis and the first scintigraphic examination was 5.83 months (Q1–Q3, 1.40–26.98 months). The median interval between initial diagnosis and first PRRT cycle was 25.06 months (Q1–Q3, 2.36–37.1 months). The median interval between initial diagnosis and the second PRRT cycle was 46.08 months (Q1–Q3, 27.73–85.2 months).

^68^Ga-DOTA-TOC and ^18^F-FDG PET/CT were performed at baseline (i.e., before PRRT), 3 months after completion of the first full PRRT course and every 6–9 months thereafter as part of a routine work-up. ^68^Ga-DOTA-TOC and ^18^F-FDG PET/CT were performed within 2 days to 6 weeks (mean 3.16 weeks) of each other. Between these two scans, no therapy including PRRT was given.

^177^Lu-DOTA-TATE were administered intravenously. The periods between baseline evaluation and the first PRRT administration, and between RECIST 1.1 assessment and PRRT administration were <4 weeks. A PRRT course included 3–4 therapies with 7.4 GBq ^177^Lu-DOTA-TATE each, administered at an interval of 10–14 weeks apart from each other.

A second full PRRT course was started after a median interval of 46.06 months (Q1–Q3, 27.75–85.2 months) after the first PRRT course. The PRRT treatment scheme was individually adapted concerning the doses and time intervals, depending on tumor stage, age, tracer uptake, biochemical response, Karnofsky Index and quality of life.

The mean cumulative administered radioactivity after the second PRRT cycle with ^177^Lu-DOTA-TATE was 48.83 ± 11.81 GBq (range 37.04–60.62 GBq).

The treatment protocol used [14] included the additional administration of “cold“, long-acting somatostatin analogues. These were administered after each ^177^Lu-DOTA-TATE injection and repeated 4 weeks thereafter. Retreatment with ^177^Lu-DOTA-TATE was performed at least 6 weeks after the use of long-acting somatostatin analogues.

### 4.3. Positron Emission Tomography (PET)

#### 4.3.1. ^68^Ga-DOTA-TOC

The preparation of ^68^Ga-DOTA-TOC was based on a fully automated synthesis, as described previously [30]. The patients received 100–150 MBq of ^68^Ga-DOTA-TOC (20–30 μg) intravenously. PET acquisition was started 60–90 min (median 75 min) after injection. Imaging was performed with a dedicated PET scanner (MS-Advance or MS-Discovery 450; GE Healthcare). Images were acquired from the head to the mid-thigh. Attenuation correction was performed using transmission data obtained with a ^67^Ge pin source at 3 min per bed position (MS-Advance) or a CT scan (MS-Discovery 450). Ordered-subsets expectation maximization was used for image reconstruction.

#### 4.3.2. ^18^F-FDG

Patients received 200–300 MBq of ^18^F-FDG intravenously after fasting for at least 8 h. PET acquisition was started 52–80 min (median 65 min) after injection. The settings and protocol were as described for ^68^Ga-DOTA-TOC.

#### 4.3.3. CT

A 2.5-mm helical CT scan was performed on a HiSpeed CT/I Advantage scanner (GE Healthcare). Approximately 1.5 mL/kg body weight of Visipaque 320 contrast medium (GE Healthcare) was administered.

#### 4.3.4. Image Review

^68^Ga-DOTA-TOC and ^18^F-FDG PET images were assessed by two experienced board-certified nuclear medicine physicians. The criteria for a positive finding on PET studies were focal area(s) of increased tracer uptake or diffusely increased uptake, excluding physiological uptake, in comparison with adjacent tissue on axial, coronal and sagittal images. When the PET results corresponded with those of conventional imaging or histopathology, or when a corresponding lesion appeared on conventional imaging during follow-up, the PET results were rated as true-positive. Lesions not detected on the PET but seen on conventional imaging and showing progression during follow-up or confirmed by histopathology were rated as false-negative. PET results suggestive of tumor lesions without corresponding lesions found unconventional imaging during follow-up or verification by histopathology were rated as false-positive. 

All PET/CT images were analyzed using commercially available software (eNTEGRA; GE Healthcare), which allowed for the review of PET, CT and fused imaging data. RECIST 1.1 was used for determining the tumor response to treatment. Based on all imaging and histological findings as appropriated, the tumor response was categorized as a complete response, partial remission, stable disease or progressive disease [31,32].

#### 4.3.5. Statistical Data Analysis and Data Collection

Patient and disease-related data were collected from hospital electronic records and imaging and from the medical reports of outside hospitals. SPSS software (version 26.0 for Windows SPSS Inc., Chicago, IL, USA, and LEAD Technologies, Charlotte, NC, USA) was used for the statistical evaluation of the results. Continuous variables are expressed as mean values with standard deviations. Graphs and tables were created using Microsoft Excel and Microsoft Word. Lifetime analyses were used to investigate the differences in the predefined groups. Kaplan–Meier graphs showed the probability of survival. The log-rank test was used to compare the survival distribution between groups. In all analyses, a p value of less than 0.05 was considered statistically significant.

## Figures and Tables

**Table 1:** Disease course after the second peptide receptor radionuclide therapy (PRRT) course.

		Partial Remission	Stable Disease	Progressive Disease	Total
Sex	Female	0	7	7	14
	Male	2	14	10	26
Total		2	21	17	40

**Table 2:** ^18^F-FDG-uptake status.

	Before PRRT	After First PRRT Course	After Second PRRT Course
Negative	33	30	26
Positive	7	10	14
Total	40	40	40

**Table 3:** Demographic data.

Characteristic	Number (n)	Percentage (%)
Total number of patients	40	
Age at initial diagnosis (years) Mean ± standard deviation Range	54.63 ± 12.85 29–83	
Gender Male Female	26 14	65.0 35.0
Primary tumor site Pancreas Stomach Small bowel Colon Rectum	18 2 18 1 1	45.0 5.0 45.0 2.5 2.5
Sites of metastases Liver Lymph nodes Bone Lung Peritoneum Small bowel Spleen Suprarenal gland	36 15 7 1 6 1 2 1	90.0 37.5 17.5 2.5 15.0 2.5 5.0 2.5
Grade 1 2 3 Unknown	2 29 8 1	5.0 72.5 20.0 2.5

n = number of patients, except age in years.
